# Indirect adjusted comparison of 6-month clinical outcomes between esketamine nasal spray and other real-world polypharmacy treatment strategies for treatment resistant depression: results from the ICEBERG study

**DOI:** 10.3389/fpsyt.2023.1250987

**Published:** 2023-10-31

**Authors:** Albino J. Oliveira-Maia, Benoit Rive, Joachim Morrens, Yordan Godinov, Jedelyn Cabrieto, Nolen Perualila, Siobhán Mulhern-Haughey

**Affiliations:** ^1^Champalimaud Research and Clinical Centre, Champalimaud Foundation, Lisbon, Portugal; ^2^NOVA Medical School, Faculdade de Ciências Médicas, NMS, FCM, Universidade NOVA de Lisboa, Lisbon, Portugal; ^3^Janssen EMEA, Paris, France; ^4^Janssen EMEA, Beerse, Belgium; ^5^Janssen EMEA, Sofia, Bulgaria; ^6^Janssen EMEA, Dublin, Ireland

**Keywords:** treatment resistant depression, real-world evidence, indirect comparison, augmentation, polypharmacy, esketamine nasal spray

## Abstract

**Background:**

The efficacy of esketamine nasal spray (NS) as a rapid-acting agent for treatment resistant depression (TRD) was demonstrated in comparisons with placebo, when both were given in addition to a newly initiated selective serotonin reuptake inhibitor (SSRI)/serotonin norepinephrine reuptake inhibitor (SNRI). How esketamine NS compares with commonly used real-world (RW) polypharmacy treatment strategies is not known.

**Method:**

ICEBERG was an adjusted indirect treatment comparison that analysed data from SUSTAIN-2 (NCT02497287; clinicaltrials.gov), a long-term, open-label study of esketamine NS plus SSRI/SNRI, and the European Observational TRD Cohort (EOTC; NCT03373253; clinicaltrials.gov), an observational study of routine clinical practice. Data were compared between patients receiving esketamine NS (SUSTAIN-2) and those from the EOTC treated with polypharmacy treatment strategies, either combination or augmentation. Analyses were adjusted for potential confounders, using rescaled average treatment effect among treated estimates. Threshold analyses were conducted to assess potential impact of unmeasured confounders on the robustness of analyses where esketamine NS was found to be significantly superior. Sensitivity analyses were used to understand the impact of analysis method selection and data handling.

**Results:**

Esketamine NS treatment resulted in a higher probability of 6-month response (49.7% [95% confidence interval (CI) 45.6–53.9]) and remission (33.6% [95% CI 29.7–37.6]) versus RW polypharmacy (26.8% [95% CI 21.0–32.5] and 19.4%, [95% CI 14.2–24.6], respectively). Relative risk calculations showed esketamine NS was 1.859 (95% CI 1.474–2.345; *p* < 0.0001) times as likely to result in response and 1.735 (1.297–2.322; *p* = 0.0002) times as likely to result in remission versus RW polypharmacy at 6 months. Threshold and extensive sensitivity analyses supported that analyses of esketamine NS superiority were robust.

**Conclusion:**

ICEBERG supports esketamine NS being superior to current RW individualized polypharmacy strategies, including augmentation, with benefits extending beyond acute use, to improved chance of 6-month response and remission. While unobserved confounding factors may certainly impact results of an indirect comparison, threshold analysis supported a low likelihood of this affecting the conclusions.

To view an animated summary of this publication, please click on the Supplementary video.

## 1. Introduction

Treatment resistant depression (TRD) is most often defined as a major depressive episode (MDE) that fails to respond to two or more different antidepressants given at adequate dose and duration (1, 2), and affects 10–30% of patients with major depressive disorder (MDD) (3–6). In the real-world (RW) setting, as many as 74% of patients with TRD do not achieve a response (i.e., 50% or greater reduction in symptom severity, as measured, for example, by the Montgomery-Åsberg Depression Rating Scale score [MADRS]) to new treatment after 6 months, and as few as 17% achieve symptom remission (defined according to minimal symptom severity, such as total MADRS score ≤ 10) (7). Importantly, the likelihood of achieving response or remission decreases as treatment failures increase (5, 8). Furthermore, relapse is common, especially in patients who achieve response but not remission (5, 7). TRD has a greater patient and societal burden than non-treatment resistant MDD, including lower health-related quality of life (HRQoL) and increased work and activity impairment (4, 9, 10). It is therefore crucial to identify which treatments are most likely to result in, and maintain, remission for patients with TRD.

A lack of evidence to support one treatment over others has resulted in a heterogenous treatment landscape for TRD. Currently, real-world treatment (RWT) involves the use of any treatment or combination of treatments approved for use in MDD (7, 11), including pharmacological monotherapy or polypharmacy (6, 11). Pharmacological monotherapy can be of any substance approved for use in MDD, prescribed on its own. Polypharmacy can be either the prescription of combinations of antidepressant medications or the augmentation of at least one antidepressant with a substance without primary antidepressant properties for MDD, such as second generation antipsychotics (e.g., aripiprazole, quetiapine and risperidone) or mood stabilizers (e.g., lithium and lamotrigine) (6, 11–14). A recent study of patients in Europe reported use of more than 50 different pharmacological medications among a cohort of 411 patients with TRD across seven European countries, with polypharmacy as the most common treatment strategy (7).

To date, almost all pharmacological antidepressant treatments target the monoamine pathway (15). In the last decade, however, newer treatments targeting glutamatergic neurotransmission have emerged as promising alternatives (16–18), including esketamine nasal spray (NS), an N-methyl-D-aspartate (NMDA) receptor antagonist (19). In randomised controlled trials (RCTs), esketamine NS in addition to either a selective serotonin reuptake inhibitor (SSRI) or a serotonin norepinephrine reuptake inhibitor (SNRI) was more effective for patients with TRD than antidepressant plus placebo (20–23). Based on these results, esketamine NS, in combination with a SSRI or SNRI, obtained American and European-wide market approval specifically for TRD (19, 24). However, only newly-initiated SSRI/SNRI monotherapy was used as the active comparator in these RCTs. Furthermore, the existing phase 3 RCTs did not combine acute and maintenance treatment phases in a single, long-term comparative study to assess esketamine NS during both treatment phases (20–23). The Indirect adjusted Comparison Estimating the Benefit of Esketamine compared with Routine treatment of TRD in General psychiatry (ICEBERG) analyses were designed to address this evidence gap. The objective was to compare long-term (6-month) data, from two distinct studies, on the efficacy of esketamine NS with that of RWT, extending both the range of comparators and period of treatment relative to currently available data.

Focusing on clinical response and remission, a parallel publication from the ICEBERG study showed patients receiving esketamine NS were almost twice as likely to achieve response or remission compared with patients receiving their physician’s best choice (RWT) (25). However, as RWT is very heterogenous (7), these findings do not guarantee superiority of esketamine NS over each different treatment type included in the mixed comparator group. Here, we focus on a more homogenous treatment strategy type, presenting adjusted comparison of response and remission rates at 6 months for patients receiving esketamine NS plus SSRI/SNRI relative to patients receiving RW polypharmacy treatment strategies. Given that there is no robust evidence to suggest higher efficacy for either combination or augmentation strategies and guidelines do not specify a preferred approach (26–28) they have been pooled in a single RW polypharmacy treatment group for the purposes of these analyses.

## 2. Methods

### 2.1. Study designs

An indirect adjusted treatment comparison (ITC) of esketamine NS with RW polypharmacy was performed using individual patient 6-month response and remission data from two studies of patients with TRD. SUSTAIN-2 (NCT02497287) was a global, long-term, single-arm, open-label study of the safety and efficacy of esketamine NS given in combination with a new oral antidepressant (SSRI or SNRI, as per the label), which included patients from Europe (29). The European Observational TRD Cohort (EOTC; NCT03373253) study was a prospective, non-interventional, multicenter study of patients initiating a new, routine treatment for TRD in RW clinical practice (7). In this RW study, all patients were receiving medication and/or other treatments according to usual care in their treatment setting, with treatment, dose and administration at the discretion of the prescribing clinician (7, 30). These studies were selected for comparison as they were designed with similar inclusion and exclusion criteria, including the same operational definition of TRD, and both provided long-term follow-up of patients. EOTC and SUSTAIN-2 study designs, along with key inclusion and exclusion criteria, are provided in Supplementary Figure S1 and the Supplementary methods, with a summary table found in Supplementary Table S1. All participants in both studies provided written informed consent.

### 2.2. Adjusted indirect treatment comparison

Data included in the adjusted ITC from the EOTC study were restricted to those from patients starting an antidepressant treatment involving at least one oral antidepressant medication. Patients that did not receive at least one antidepressant medication (e.g., receiving an antipsychotic as monotherapy, or receiving only neurostimulation treatments and/or psychosocial interventions without an accompanying antidepressant) were excluded. No patients in the EOTC received esketamine NS as it was not available to prescribe until after the study ended. The analyses reported here focused on patients from the EOTC who were on a polypharmacy treatment strategy. This included any patient taking at least one oral antidepressant (a) combined with one or more additional oral antidepressant (combination therapy) and/or (b) augmented with one or more antipsychotic or mood stabilizing substances (augmentation therapy). Handling of dropouts and treatment changes is described in Supplementary methods and Supplementary Table S2.

### 2.3. Covariates for adjustment

Some level of imbalance was expected between the two treatment groups, as patients were not randomly assigned to one or the other of the studies. It is possible that the cohorts may have had different baseline prognostic factors leading to confounding effects on the outcomes and bias in favor of one treatment. Patient covariates, reported in both SUSTAIN-2 and the EOTC studies, covering sociodemographics as well as clinical, psychometric, disease and treatment history (Supplementary Table S3), were used in analyses comparing data from the two studies.

### 2.4. Main analyses

Esketamine NS was compared to polypharmacy using two different approaches to adjust for imbalances between both study populations, based on potential prognostic factors. Both approaches used 17 baseline patient covariates that both SUSTAIN-2 and the EOTC data had in common. For the main analysis, propensity score (PS) based inverse probability weighting (IPW) comparisons were used. Data from patients in the RW polypharmacy arm (EOTC) were reweighted using a rescaled average treatment effect among treated (ATT) IPW method. This approach, based on propensity scores estimated using the 17 patient covariates, aimed to remodel the EOTC data to act as a matched RW polypharmacy pseudo-control arm for the data from SUSTAIN-2. Data from patients on RW monotherapy were also reweighted using ATT IPW for comparison purposes with the main analyses. The covariates and IPW are described in more detail in the Supplementary methods.

Response to treatment (≥50% improvement in total MADRS score, relative to baseline) and remission (total MADRS score ≤10) at 6 months were compared between the two studies. Analysis was based on observed cases and treatment effect was calculated as an odds ratio (OR). For illustrative and interpretation purposes, values for relative risk (RR), risk difference (RD) and number needed to treat (NNT), as well as the estimated probability of achieving response or remission, were also produced. Threshold analyses were carried out when esketamine NS was significantly superior, to assess how much lower the rates of response and remission for esketamine NS could be without losing statistically significant superiority over RW polypharmacy (Supplementary methods).

### 2.5. Multivariable analyses

The second approach used multivariable logistic regression models to compare esketamine NS with RW polypharmacy while accounting for potential between-study imbalances in the distribution of the 17 covariates. Using the adjusted OR, the models were also used to identify the variables that were the strongest predictors of response and remission.

### 2.6. Sensitivity analyses

Sensitivity analyses (SA), examining the effect of using either different IPW methods (SA1, SA2 and SA3) or different data handling approaches (SA4 and SA5) are described in Supplementary methods.

## 3. Results

### 3.1. Baseline characteristics

Study flow diagrams detailing inclusion of patients from the SUSTAIN-2 and EOTC studies are shown in Supplementary Figure S2. Before reweighting, baseline characteristics of patients in the esketamine NS group (*n* = 559) were largely comparable to those in the RW polypharmacy group (*n* = 225) including the percentage of women, mean age, mean number of treatment failures in the current MDE and mean duration of each treatment received during the current MDE (Table 1).

**Table 1:** Baseline characteristics.

Category Mean (SD) unless otherwise stated	Esketamine NS (*N* = 559)	RW polypharmacy (*N* = 225)
Age, years	49.8 (12.7)	51.4 (10.3)
Gender, % (*n*)		
Female	63.5 (355)	62.2 (140)
Age at diagnosis, years	35.0 (13.4)	37.6 (13.1)
Time since first diagnosis of MDD, years	14.7 (11.4)	13.8 (11.4)
Total MADRS score	31.2 (5.0)	32.2 (6.0)
Total number of failures in current episode	2.6 (1.0)	2.7 (1.0)
CGI-S score	4.8 (0.7)	4.8 (0.8)^a^
EQ-VAS score	44.4 (19.8)	40.6 (18.1)^b^
Total number of MDE	4.1 (3.3)^c^	4.1 (4.4)^c^
Duration of current MDE, years	2.5 (4.2)	2.4 (2.8)
History of suicidality (based on C-SSRS; lifetime), % (*n*)		
No event	61.2 (342)	44.4 (100)
Suicidal ideation	23.4 (131)	30.2 (68)
Suicidal behavior	15.4 (86)	8.9 (20)
Data missing	0	16.4 (37)
Average duration of each treatment line during current MDE, weeks^d^	43.2 (68.6)	46.6 (51.6)
Prior failure on augmentation drug, % (*n*)	15.9 (89)	15.6 (35)
Prior failure on SSRI, % (*n*)	75.1 (420)	82.2 (185)
Prior failure on SNRI, % (*n*)	50.1 (280)	55.1 (124)
Prior failure on TCA, % (*n*)	7.9 (44)	17.8 (40)
Prior failure on ‘other’ treatment,^e^ % (*n*)	51.9 (290)	50.7 (114)

^a^CGI-S data were missing from one patient on RW polypharmacy; ^b^EQ-VAS data were missing for five patients on RW polypharmacy; ^c^Data on the number of MDE were missing for one patient on esketamine NS and four patients on RW polypharmacy; ^d^Every patient received multiple treatment lines during their current MDE and these data are the average duration of each individual treatment line; ^e^‘Prior failure on other treatment’ includes trazodone, nefazodone, bupropion, mirtazapine, mianserin, opipramol, agomelatine, tianeptine, reboxetine, vilazodone and vortioxetine. CGI-S, Clinical Global Impression-Severity; C-SSRS, Columbia-Suicide Severity Rating Scale; EQ-VAS, EuroQoL-visual analog scale; MDD, major depressive disorder; MDE, major depressive episode; NS, nasal spray; RW, real-world; SD, standard deviation; SNRI, serotonin-norepinephrine reuptake inhibitor; SSRI, selective serotonin reuptake inhibitor; TCA, tricyclic antidepressant.

### 3.2. Performance of PS reweighting of comparator group data

PS reweighting of the baseline covariates showed a larger overlap in distributions after reweighting. Following reweighting, standardised mean difference (SMD) between polypharmacy and esketamine NS treatment was reduced across almost all baseline variables (Supplementary Figure S3). Indeed, all SMDs were between −0.2 and + 0.2, indicating that none were clinically detectable. In exploratory analyses, PS reweighting was also applied to data from 82 patients treated with monotherapy at baseline, for potential comparison with the main analyses. However, in this subgroup, reweighting did not reduce the SMD between treatments for any of the variables in the full, 17 covariate model (Supplementary Figure S4). Including fewer covariates also did not lead to reductions in the SMDs (data not shown), so no further analysis was feasible in the monotherapy subgroup.

### 3.3. Probabilities of response and remission (IPW, ATT)

**Figure 1:**
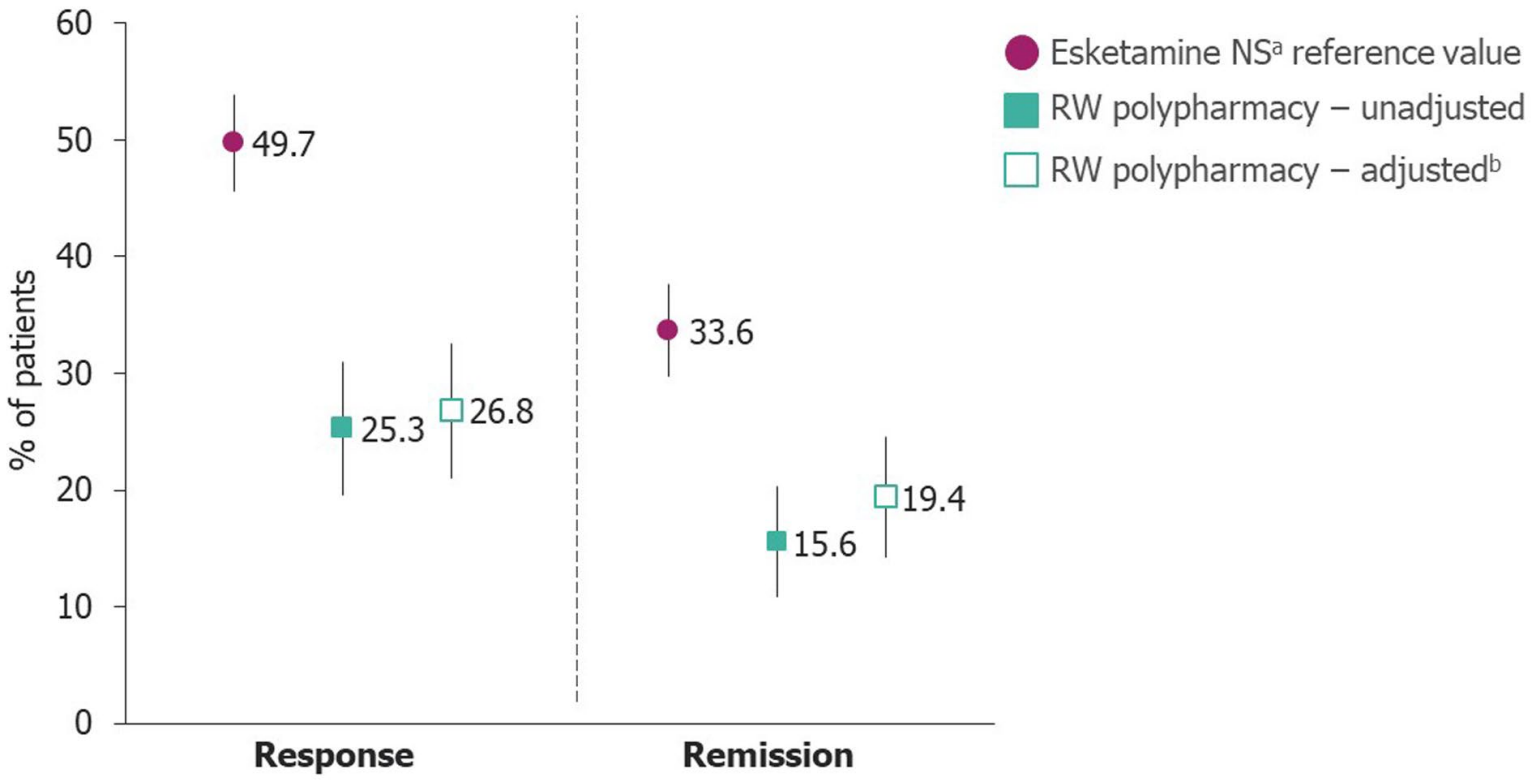
Probability of response and remission at Month 6. ^a^Given in combination with an SSRI or SNRI; ^b^RW polypharmacy data were adjusted using the ATT-cumulative covariate adjustment method. Error bars represent upper and lower CIs. ATT, rescaled average treatment effect among treated; CI, confidence interval; NS, nasal spray; RW, real-world; SNRI, serotonin norepinephrine reuptake inhibitor; SSRI, selective serotonin reuptake inhibitor.

In unadjusted analyses of data observed at Month 6, response was reached in 278/559 (49.7%) of patients taking esketamine NS. In patients receiving RW polypharmacy, 57/225 (25.3%) reached response and following ATT reweighting, the estimated probability of response was 26.8% (95% confidence interval [CI] 21.0–32.5%; Figure 1). The OR (95% CI) of achieving response with esketamine NS versus RW polypharmacy was 2.709 (1.930–3.802; *p* < 0.0001; Table 2). Significant superiority of esketamine NS in achieving response was also found when other treatment effect measures were estimated. In terms of RR, patients taking esketamine NS were 1.859 (1.474–2.345; *p* < 0.0001) times as likely to achieve response than patients on RW polypharmacy. RD (95% CI) values represented an additional 23.0% (15.9–30.1%) of patients achieving response with esketamine NS compared with RW polypharmacy. When NNT values were considered, five patients would need to be treated with esketamine NS and five with RW polypharmacy to obtain one additional patient experiencing response in the esketamine group relative to the RW polypharmacy group.

**Table 2:** Relative chances of response and remission at Month 6.

	Esketamine NS^a^ *vs* RW polypharmacy (95% CI); *p* value
**Response**	
OR	2.709 (1.930–3.802); <0.0001
RR	1.859 (1.474–2.345); <0.0001
RD	0.230 (0.159–0.301); <0.0001
NNT	5 (4–7)
**Remission**	
OR	2.108 (1.449–3.067); 0.0001
RR	1.735 (1.297–2.322); 0.0002
RD	0.143 (0.078–0.207); <0.0001
NNT	8 (5–13)

RW polypharmacy data were adjusted using the ATT covariate adjustment method. ^a^Given in combination with an SSRI or SNRI. OR > 1 indicates esketamine is superior to the comparator treatment. ATT, rescaled average treatment effect among treated; CI, confidence interval; NNT, number needed to treat; NS, nasal spray; OR, odds ratio; RD, risk difference; RR, relative risk; RW, real-world; SNRI, serotonin-norepinephrine reuptake inhibitor; SSRI, selective serotonin reuptake inhibitor.

In unadjusted analyses, remission was reached by 188/559 (33.6%) patients receiving esketamine NS and 35/225 (15.6%) patients receiving RW polypharmacy at Month 6. Following ATT IPW adjustments, the estimated probability (95% CI) of remission for esketamine NS was 33.6% (29.7–37.6%) and 19.4% (14.2–24.6%) in the RW polypharmacy group (Figure 1). The OR (95% CI) of achieving remission with esketamine NS versus RW polypharmacy was 2.108 (1.449–3.067; *p* = 0.0001; Table 2). Regarding RR, patients taking esketamine NS were 1.735 (1.297–2.322, *p* = 0.0002) times as likely to achieve remission than patients on RW polypharmacy. RD (95% CI) values represented an additional 14.3% (7.8–20.7%) of patients achieving remission on esketamine NS compared with RW polypharmacy. NNT values showed that eight patients need to be treated with esketamine NS so that one more patient would achieve remission relative to the numbers obtained with RW polypharmacy.

The main analysis used the ATT IPW adjustment approach to reweighting. Three sensitivity analyses using alternative IPW approaches (average treatment effect among control [ATC; SA1], stabilized average treatment effect [sATE; SA2] and average treatment effect among the overlap population [ATO; SA3]) also demonstrated superiority of esketamine NS over RW polypharmacy (Supplementary Tables S4, S5). To assess the impact of alternate approaches to the handling of dropouts and other sources of missing data, two further SAs were conducted, designed to be less conservative regarding inclusion in the esketamine NS group (SA4), or more conservative regarding treatment changes in the RW polypharmacy group (SA5). Baseline characteristics of the resulting SA treatment groups are shown in Supplementary Table S6. Neither SA altered the conclusion that esketamine NS was superior to RW polypharmacy, with SA5 actually suggesting a greater benefit from esketamine NS (Supplementary Tables S4, S5). Thus, the results for response and remission at Month 6 were significantly in favor of esketamine NS, across all reweighting adjustments in the RW polypharmacy group data and sensitivity analyses conducted.

Threshold analysis showed the maximum loss of absolute response rate that could occur in patients receiving esketamine NS before loss of significance (*p* > 0.05) in comparison with RW polypharmacy ranged from 15.4–15.9%, depending on efficacy measure. The equivalent threshold value for remission was 7.3–7.8% (Supplementary Table S7).

### 3.4. Multivariable analysis of response and remission

Multivariable analysis is presented in Supplementary Figure S5. Treatment with esketamine NS was the largest predictor of response and remission. Age at MDD diagnosis of ≥55 years was strongly associated with a reduced chance of response. Baseline MADRS >34, as well as prior failure on augmentation, SNRI, tricyclic antidepressant (TCA) or ‘other’ treatment (trazodone, nefazodone, bupropion, mirtazapine, mianserin, opipramol, agomelatine, tianeptine, reboxetine, vilazodone and vortioxetine) were also associated with a reduced chance of response. Baseline MADRS ≥31, prior failure on augmentation and prior failure on ‘other’ treatment were all associated with a reduced chance of remission.

## 4. Discussion

It is widely acknowledged that TRD is a difficult condition to treat (6, 11, 13, 31). Despite the large number of treatments available for MDD and an even greater number of combinations that can be devised and tailored to each patient, response or remission is not achieved in most patients with TRD (7). With no clear evidence to date for any one pharmacological treatment being better than others, patients with TRD may remain unwell for long periods of time while multiple different medications and combinations of medications are prescribed and failed (7, 30). The indirect comparison presented here provides support for the superiority of esketamine NS over RW polypharmacy strategies used in Europe, including combinations of several antidepressants and augmentation of an oral antidepressant with medications such as antipsychotics and mood stabilizers. In fact, when compared with those receiving RW polypharmacy treatment, the chance of response to treatment in patients with TRD treated with esketamine NS relatively increased by 86%, while the likelihood of remission was relatively increased by 74%. Such an increase in remission rate may be of even greater value in the long-term, since relapse is less likely in patients with TRD who achieve remission rather than just response (32, 33).

### 4.1. Assessment of methodological robustness

The methods described here are widely accepted and recommended in the absence of a direct comparison (34–38). A more detailed discussion of the methods employed in this study was provided in the first analysis of these data (25), a key strength being the use of individual patient level data. Congruent conclusions across all methods and sensitivity analyses support the robustness of the main analysis.

Although the population characteristics of the two studies used in this comparison were broadly similar, it was fundamental to rule out potential bias in favor of one of the two populations due to differences in baseline characteristics. Thus, PS reweighting, using covariates corresponding to baseline patient characteristics, was used to create a well-matched pseudo control arm for the ITC analyses. These same covariates were added to the ITC models, resulting in outputs that were adjusted for potential confounding factors. Importantly, results from adjusted versus unadjusted comparisons were largely similar, confirming that treatment effect differences were not substantially biased by differences in the characteristics of the two study populations.

Threshold analyses were conducted to measure the margin by which the remission or response rate might theoretically be lost in the esketamine NS group, while retaining significant superiority over RW polypharmacy. A 15.4% loss of absolute response rate and a 7.3% loss of remission rate in patients receiving esketamine NS would be possible without losing significance in the superiority over RW polypharmacy. This analysis can also be used to measure how much a hypothetical, unobserved confounder might be contributing to artificially overestimate response or remission rates in the esketamine NS arm in the absence of significant differences relative to RW polypharmacy. Indeed, if there was a hypothetical unobserved confounder that had increased response rates by 50% and was 30% more prevalent in the SUSTAIN-2 population than the EOTC population, this could have artificially overestimated the esketamine response rate by 15% (50% x 30%) in SUSTAIN-2. However, even if such an overestimate was adjusted for, superiority of esketamine NS versus RW polypharmacy would still be statistically significant, as 15% is below the estimated margin (15.5%) calculated in the threshold analysis. Similarly, for remission, a hypothetical unobserved confounder that had increased remission rates by 35% and was 20% more prevalent in the SUSTAIN-2 population than the EOTC population, could result in an artificial overestimation of the esketamine remission rate by 7% (35% x 20%) in SUSTAIN-2. However, even after adjusting for such an overestimate, the superiority of esketamine NS versus RW polypharmacy would still be statistically significant, as 7% is below the estimated margin (7.5%) calculated in the threshold analysis. In any case, it is unlikely that unobserved confounders with such high levels of prevalence and impact exist.

### 4.2. Long-term benefits of esketamine NS

This study is the first to generate data on the long-term efficacy of esketamine NS compared with routinely used polypharmacy treatment strategies and, as such, adds substantially to currently available evidence (39). Short-term (3 months) RWE data are available in country-specific studies, such as the REAL-ESK study, and show a significant reduction in depressive symptoms (40). In the ICEBERG analysis, after 6 months of treatment, esketamine NS showed significant benefit over other polypharmacy strategies. Although the efficacy of esketamine NS has been demonstrated in the context of a rapid-acting acute phase TRD treatment (20–23, 29, 41), longer-term use of esketamine NS may be of further benefit to patients to avoid relapse. The SUSTAIN-1 clinical trial examined the effect of withdrawing esketamine NS treatment (20). After 16 weeks on esketamine NS, stable remitters and responders were randomised to either continue or switch from esketamine NS to placebo NS. The risk of relapse was both substantially and significantly greater in patients who stopped esketamine NS treatment compared with those who continued. Furthermore, the relapse risk was greater in patients who had only achieved response, rather than remission, highlighting the importance of achieving remission for patients with TRD.

### 4.3. Polypharmacy as a subgroup comparator

The results from the ICEBERG polypharmacy analyses confirmed previously published results that found numerically larger treatment effects with esketamine NS versus placebo compared with second-generation antipsychotics versus placebo (14). The polypharmacy methodology reported here allowed for further testing of the differences between esketamine NS and polypharmacy treatment. The large sample size of both the EOTC and SUSTAIN-2 studies was an important strength of these analyses. Despite creating a smaller, strategy-specific treatment dataset (polypharmacy) from the RWT group, the resulting sample size was still adequate to include all the key medically relevant variables (covering sociodemographics, treatment and disease history, and baseline clinical and patient-reported scales) in the adjustments.

The rationale for pooling combination and augmentation to create a polypharmacy subgroup population was based on several considerations. First, current treatment guidelines consistently recommend that escalation strategies after failure of second-line treatment include combination or augmentation strategies as evidence-based therapeutic approaches in TRD (28, 42, 43). However, guidelines do not specify the treatment line deemed most appropriate for initiation of either strategy (26). Second, selection of treatment options for patients with TRD beyond second-line treatment is highly individualized, taking into account previous treatment history, comorbidities and current concomitant medications, as well as each patient’s circumstances and treatment preferences (28, 43). Thus, there is no guidance for choosing either combination or augmentation strategy in any specific patient population. Third, both augmentation and combination treatment strategies are significantly associated with severe depression symptoms, high psychiatric burden, treatment resistance and high levels of comorbidities (44). Patients in these treatment groups are likely to be more complex than patients on monotherapy, having reached these types of treatments *via* stepwise treatment escalation, but are not necessarily more complex than each other. In summary, there is no robust evidence to suggest that either combination or augmentation therapy is more effective than the other, and this is reflected in the treatment-agnostic approach set out in the current guidelines to select between one or the other (28, 42, 43).

Ideally, a direct and randomised comparison of esketamine NS with all other treatments and combinations used in routine clinical practice would be conducted. However, such is the heterogeneity of treatments prescribed to patients with TRD (7), that it would be prohibitively complicated to design such an interventional study with the numbers of patients needed to statistically power each individual comparison. An indirect treatment comparison using data from two well-aligned studies was therefore considered as a best-in-class alternative approach, albeit still restricted by the heterogeneity of the RW polypharmacy data. The EOTC patients, together, reported over 50 different pharmacological treatments at baseline (7), with the top five treatments representing only 40% of the medications reported by patients, and thus not representing the majority. To analyse individual treatments and combinations in the EOTC would result in too small sample sizes; this would hinder the inclusion of relevant adjustment variables into the statistical regression model (PSs) and leave the results vulnerable to potential confounders.

When data from patients on monotherapy were analysed, PS reweighting failed to reduce the mean difference between the treatments across any of the variables, even when fewer variables were included in the model. The size of the monotherapy population was thus insufficient to provide a reliable adjusted comparison for a robust ITC analysis and polypharmacy treatment is the smallest comparator subgroup for which ITC analysis is presented. This polypharmacy grouping may have masked differences in efficacy between esketamine NS and individual pharmacological treatments that may otherwise have been apparent. However, the high degree of additional benefit of esketamine NS over polypharmacy adds confidence to the conclusions drawn. Furthermore, such a stratification by treatment strategy type (i.e., separating out the polypharmacy subgroup) resulted in a more homogenous pool of treatments than when analysing the mixed RWT group as a whole (i.e., patients receiving any pharmacological treatment). Stratification therefore provided a good trade-off between the homogeneity of the comparator and the ability to adjust for potential confounders, optimizing the unbiased estimation of treatment effect.

### 4.4. Limitations

As in any non-randomised comparison, residual confounding due to unobserved prognostic factors cannot be ruled out. However, most clinically important variables were taken into account in these analyses. It is possible that study-related factors aside from the specific medications received may have differentially influenced response and remission rates in the esketamine NS group compared with the RW polypharmacy group. For example, motivation and compliance of patients in the SUSTAIN-2 trial may have been higher due to the nature of clinical trial management. Furthermore, patients in SUSTAIN-2 had more frequent clinic visits than those in the EOTC, since healthcare professionals were required to directly supervise administration of esketamine NS, and the impact of increased contact with healthcare professionals in this context is unknown. However, threshold analyses suggest that, to change the conclusion regarding the significant benefit of esketamine NS over RW polypharmacy, the impact of such study-related factors would need to be substantial.

### 4.5. Future directions

An open-label randomised study to compare esketamine NS with extended-release quetiapine (ESCAPE-TRD; NCT04338321) will provide additional comparative evidence (45). Quetiapine is an augmentation agent that is recommended as an add-on treatment in patients with MDD who have had a suboptimal response to treatment with other antidepressants (46). In the interim, this ITC provides data supporting the benefit of esketamine NS over a very diverse set of polypharmacy treatment strategy types.

## 5. Conclusion

This indirect treatment comparison suggests esketamine NS is beneficial over the RW polypharmacy strategies currently used in general psychiatry for treatment of TRD. This evidence is robust, and indicates that the benefit extends beyond acute use, with substantial improvements in the chances of achieving remission over other treatment strategies after 6 months. Esketamine NS, as a more effective alternative to existing RW polypharmacy strategy types, may provide a clearer treatment path for patients in an otherwise heterogenous treatment landscape, and thus increase their chances of achieving remission quickly.

## Animated summary

To view an animated summary of this publication, please click on the Supplementary video, or visit the manuscript online at: https://doi.org/10.3389/fpsyt.2023.1250987.

## Data availability statement

The raw data supporting the conclusions of this article will be made available by the authors, without undue reservation.

## Ethics statement

Ethical approval was not required for the studies involving humans because as this publication reports findings from an indirect treatment comparison, ethical approval was independently obtained from the primary studies. Further information regarding ethical approval can be found in the respective primary publications. The studies were conducted in accordance with the local legislation and institutional requirements. Written informed consent for participation was not required from the participants or the participants’ legal guardians/next of kin in accordance with the national legislation and institutional requirements because as this publication reports findings from an indirect treatment comparison, written informed consent from patients was not required for this analysis.

## Author contributions

AJOM, BR, JM, YG, JC, NP, and SMH contributed to study conception, design, analysis, interpretation of the data, and drafting the article or revising it critically for important intellectual content. All authors contributed to the article and approved the submitted version.
