# Supplementary material for: Indirect adjusted comparison of 6-month clinical outcomes between esketamine nasal spray and other real-world polypharmacy treatment strategies for treatment resistant depression: results from the ICEBERG study
Source: Front Psychiatry. 2023 Oct 31;14:1250987. doi: 10.3389/fpsyt.2023.1250987 (PMC10669145; doi:10.3389/fpsyt.2023.1250987)
Supplement: Supplementary file 2 [file Data_Sheet_1.docx]

Supplementary Material

Indirect Adjusted Comparison of 6-Month Clinical Outcomes Between Esketamine Nasal Spray and Other Real-World Polypharmacy Treatment Strategies for Treatment Resistant Depression: Results from the ICEBERG Study

Oliveira-Maia AJ,^1,2^ Rive B,^3*^ Morrens J,^4^ Godinov Y,^5^ Cabrieto J,^4^ Perualila N,^4^ Mulhern‑Haughey S^6^

*** Correspondence:** Benoit Rive: brive@its.jnj.com

# Supplementary Methods

## SUSTAIN-2 and EOTC study inclusion criteria

| **SUSTAIN-2** | **EOTC** |
| --- | --- |
| Patients ≥18 years | Patients ≥18 years |
| No upper age limit | ≤74 years |
| Baseline Montgomery-Åsberg Depression Rating Scale (MADRS) score ≥22 | Baseline MADRS score ≥20 |
| Diagnosis of major depressive disorder (MDD) without psychotic features, according to the Fifth Edition of the Diagnostic and Statistical Manual of Mental Disorders (DSM-5) (American Psychiatric Association, 2013) or the Tenth Revision of the International Statistical Classification of Diseases and Related Health Problems (ICD-10) (World Health Organisation, 2004) | |
| Met the criteria for treatment resistant depression (TRD), defined as failure to respond to antidepressant treatment (i.e., ≤25% improvement of depressive symptoms when treatment was working at its best) for ≥two pharmacological treatments of sufficient duration (≥6 weeks) and dosage in the current major depressive episode (MDE) documented in the Massachusetts General Hospital–Antidepressant Treatment Response Questionnaire (MGH-ATRQ) | |

Further details of inclusion and exclusion criteria for SUSTAIN-2 and the EOTC study have been published previously (Heerlein et al., 2021; Wajs et al., 2020). DSM-5: Diagnostic and Statistical Manual of Mental Disorders 5^th^ Edition; EOTC: European Observational TRD Cohort; ICD-10: International Statistical Classification of Diseases and Related Health Problems 10^th^ Edition; MADRS: Montgomery-Åsberg Depression Rating Scale; MDD: major depressive disorder; MDE: major depressive episode; MGH-ATRQ: Massachusetts General Hospital-Antidepressant Treatment Response Questionnaire; TRD: treatment resistant depression.

## ICEBERG analysis data inclusion criteria and data handling

All direct-entry patients from SUSTAIN-2 who met the baseline selection criteria were included in ICEBERG. Patients who were transferred from the TRANSFORM-3 phase 3 trial to SUSTAIN-2 had already received four weeks of treatment with either esketamine NS or placebo NS, both given with an oral antidepressant. This meant they did not initiate esketamine NS and oral antidepressant as new treatments at SUSTAIN-2 baseline, unlike direct entry patients, and were therefore excluded from analysis (**Supplementary Figure 1a**).

Response and remission outcomes were based on observed data obtained at 6 months. However, 6-month remission and response data were not collected in some situations. Dropouts from SUSTAIN-2, by definition, stopped esketamine NS treatment; a non‑responder imputation (NRI) approach was therefore applied, considering these patients (including Week 4 non-responders who stopped treatment as per protocol) as non-responders/non-remitters. Importantly, in SUSTAIN-2, only patients achieving treatment response at Week 4 continued in the study. However, all patients were included in the ICEBERG analysis set, including those experiencing treatment failure at Week 4 for whom an NRI at 6-months was used. Furthermore, SUSTAIN‑2 was terminated when at least 300 patients had received esketamine NS for 6 months and at least 100 patients had received esketamine NS for 12 months. On reaching this point, the study stopped before 6 months for a number of the remaining patients and ‘study terminated by sponsor’ was recorded. For this reason, only patients from SUSTAIN-2 with adequate follow-up for Month 6 evaluation were included in the main ITC analysis.

The EOTC study was terminated after the last enrolled patient reached the 6‑month follow‑up visit, so unlike SUSTAIN-2 there were no patients for whom the study was terminated by sponsor. In contrast with SUSTAIN-2 and to take a conservative approach to the analyses, data from EOTC patients who dropped out (for any reason) did not have an NRI approach applied and were not included in any analyses. At the discretion of their treating physician, patients in the EOTC could switch treatment or add an antidepressant or augmentation drug to their original antidepressant and remain in the study, reflecting the flexibility of RWT for TRD. In the main analysis, an outcome of response or remission at Month 6 for patients in the EOTC counted as a positive outcome, regardless of the number of treatment changes that might have occurred between baseline and Month 6, each of which would be suggestive of treatment failure. By contrast, any treatment failure occurring in SUSTAIN-2 before Month 6 would be counted as a negative outcome, ensuring a conservative approach to comparing esketamine NS with RW polypharmacy.

## Covariates for adjustment and PS reweighting

Inverse probability weighting (IPW) was applied to balance both cohorts on all available baseline patient and disease characteristics. Data on several covariates (17 in total, covering patient characteristics including sociodemographics, clinical, psychometric, disease and treatment history) were collected in both the SUSTAIN-2 and EOTC studies (**Supplementary Table 2**). Coded categories were used for continuous covariates to avoid preconceptions regarding the nature of associations and to allow for any type of association with the tested outcomes. The covariates were ranked, based on internal medical expertise, and included in the analyses sequentially (in rank order) and cumulatively; analyses presented here are restricted to the final models, including all variables.

Propensity scores were calculated based on multivariable logistic regression, and then transformed into weights. The 17 covariates were used in a PS weighted comparison to estimate the probability of receiving esketamine NS (SUSTAIN-2) or RW polypharmacy (EOTC). Using SUSTAIN-2 as a reference, unbiased treatment differences were estimated by reweighting observations in the EOTC (IPW using PSs). The remodelled EOTC data were thus able to be used as a pseudo-control arm for SUSTAIN-2, corresponding to an average treatment effect among treated (ATT) approach. Weights were re-scaled (ATT) to correspond to the original number of patients, to prevent the sample size from becoming artificially inflated. Results of the comparison were expressed as predicted probabilities for each treatment, odds ratios (OR), relative risk (RR) and risk differences (RD), including 95% CIs. Outputs were estimated using weighted logistic regression. Number needed to treat (NNT; derived from the RD) was also reported. To assess the ability of reweighting to reduce potential imbalances between SUSTAIN-2 and the EOTC, we compared the weighted distribution of PS of the reweighted populations and the standardised mean difference (SMD) of each covariate between the two studies, before and after reweighting.

## Sensitivity and threshold analyses

To assess how robust conclusions from the main analysis were, five sensitivity analyses (SAs) were performed.

Alternative IPW methods were used to produce different estimates of treatment effect in different pseudo populations, namely stabilised average treatment effect (sATE, SA1), rescaled average treatment effect among control (ATC, SA2) and average treatment effect among the overlap population (ATO, SA3).

A further two SAs were performed using the same PS re-weighting method as the main analysis (ATT) but alternative data handling approaches (**Supplementary Table 1**). In SA4, patients who enrolled in SUSTAIN-2 less than 6 months before study termination were re‑included in the analysis, using a last observation carried forward (LOCF) approach if they withdrew due to study termination; if they withdrew for any other reason they were considered to be non-responders (NRI).

In SA5, patients in the EOTC who switched from, combined or augmented their original baseline treatment were considered as treatment failures. Data from EOTC dropouts were treated in the same way as for all other analyses and were excluded in this SA.

Threshold analyses were conducted in cases where differences between SUSTAIN-2 and EOTC were statistically significant. Threshold analysis was performed using observed results in simulations, whereby the response or remission rate in the esketamine NS arm (SUSTAIN-2) was progressively decreased without altering the rate in the RW polypharmacy arm. With each decrease, the main analyses (OR, RR and RD) were re-run to check statistical significance (p<0.05) and the process repeated until significance was lost. Differences between observed and simulated results were calculated to ascertain how much lower response and remission rates could have been in the esketamine NS arm while still showing statistically significant superiority compared with RW polypharmacy.

# Tables and Figures

## Supplementary Tables

### Supplementary Table 1. Summary of methodologies in the EOTC and SUSTAIN-2

|  | | **EOTC** | | **SUSTAIN-2** | |
| --- | --- | --- | --- | --- | --- |
| **Eligibility criteria** | | - ≥18 years - Diagnosis of MDD without psychotic features - Non-response to ≥2 antidepressants in the current episode - MADRS ≥22 at screening | | | |
| **Treatment strategy** | | Patients initiated a new, routine treatment for TRD. Monotherapy, combination therapy and augmentation therapy were all allowed, and patients could switch treatments and continue in the study. | | 56/84mg esketamine NS 2x weekly for four weeks, then weekly/every two weeks all alongside a newly initiated SSRI/SNRI | |
| **Follow-up (frequency, inpatient, outpatient)** | | Baseline data collection and a 12-month observational period with a minimum follow-up of approximately 6 months for each enrolled patient, plus an extended observation period up to 6 months from recruitment of the last patient | | Re-evaluation at four‑week intervals | |
| **Relevant outcome** | | Change in MADRS total score | | | |

IPTW: inverse probability of treatment weighting; MADRS: Montgomery-Åsberg Depression Rating Scale; MDD: major depressive disorder; NS:‍ nasal spray; SNRI: serotonin‑norepinephrine reuptake inhibitor; SSRI: selective serotonin reuptake inhibitor; TRD: treatment resistant depression.

### Supplementary Table 2. Data imputation (main analysis and sensitivity analyses)

|  | **Main analysis,**  **SA1, SA2 and SA3** | **SA4** | SA5 |
| --- | --- | --- | --- |
| Treatments prescribed at baseline in EOTC | ≥1 pharmacological treatment | ≥1 pharmacological  treatment | ≥1 pharmacological treatment |
| Data available at Month 6 |  |  |  |
| EOTC | Observed data | Observed data | Observed data |
| SUSTAIN-2 | Observed data | Observed data | Observed data |
| Data missing due to drop‐out before Month 6^a^ |  |  |  |
| EOTC | Not included | Not included | Not included |
| SUSTAIN-2 | NRI | NRI | NRI |
| Data missing due to enrolment <6 months before study termination^b^ |  |  |  |
| EOTC | N/A | N/A | N/A |
| SUSTAIN-2 | Not included | LOCF if discontinued due to study termination, NRI if discontinued for any other reason | Not included |
| Data from patients whose treatment was switched/combined/augmented |  |  |  |
| EOTC | Observed data | Observed data | NRI |
| SUSTAIN-2 | N/A | N/A | N/A |

^a^In SUSTAIN-2, any patient stopping any part of their medication was dropped from the study; ^b^The end of SUSTAIN-2 occurred when ≥300 and ≥100 patients had received esketamine NS for 6 and 12 months, respectively. When this point was reached, all patients still in the study were withdrawn, with “study terminated by sponsor” cited as reason for withdrawal. No patient selection was based on Week 4 evaluation; all patients from SUSTAIN-2 that met selection criteria were included. SUSTAIN-2 patients stopping for any other reason were imputed as NRI, even if included too close to study termination to ever reach the 6-month visit. EOTC: European Observational TRD Cohort; LOCF: last observation carried forward; NRI: non-responder imputation; NS: nasal spray; TRD: treatment resistant depression.

### Supplementary Table 3. Covariates, ranked by expert medical opinion, used for PS inverse probability reweighting (IPW) and logistic regression model adjustment comparison

| Rank | Covariate | **Categorisation** | **Notes** |
| --- | --- | --- | --- |
| 1 | Total number of failures in  current MDE | 2/3/≥4 | Based on MGH-ATRQ |
| 2 | Age, years | <30/30–44/45–64/≥65 | Data cut-off for ‘elderly patients’ at ≥65, other cut-offs determined by exploratory analysis |
| 3 | MADRS score, baseline | <31/31–34/>34 | Data cut-off >34 to identify patients with severe TRD (Muller et al., 2003), cut-off <31 determined by exploratory analysis |
| 4 | Total number of MDE | 1/2–5/6–10/>10 | Based on medical history |
| 5 | Duration of current MDE, weeks | ≤32/33–51/52–103/≥104 | Based on medical history; data cut-offs of 33 and 52 weeks correspond to quartiles of distribution, cut-off of 104 weeks based on SUSTAIN-2 study protocol which stated ‘if single episode MDD, the duration must be ≥2 years’ |
| 6 | Gender | Male/Female |  |
| 7 | Prior failure on augmentation | No/Yes | Based on MGH-ATRQ |
| 8 | History of suicidality | No event/Suicidal ideation/Suicidal behaviour/Missing | Based on C-SSRS (lifetime) |
| 9 | Time since first diagnosis of MDD, years | <5/5–19/≥20 | Based on medical history; data cut-offs determined by exploratory analysis |
| 10 | Age at diagnosis of MDD, years | <35/35–54/≥55 | Based on medical history; data cut-off of 55 corresponds to previously conducted post-hoc analysis of ‘late onset’ patients from TRANSFORM-3, cut-off of 35 determined by exploratory analysis |
| 11 | Prior failure on SSRI | No/Yes | Based on MGH-ATRQ |
| 12 | Prior failure on SNRI |  |  |
| 13 | Prior failure on TCA |  |  |
| 14 | Prior failure on other treatment^a^ |  |  |
| 15 | Average duration of each treatment line during current MDE, weeks | <12/12–23/24–51/≥52 | Every patient received multiple treatment lines during their current MDE, these data represent average duration of each individual treatment line and not overall duration of current MDE, data were calculated as duration of current MDE (based on medical history) divided by total number of failures in current MDE (based on MGH-ATRQ); data cut-offs correspond to quartiles of distribution |
| 16 | CGI-S score, baseline | 1–4/5/6–7/Missing |  |
| 17 | EQ-VAS score, baseline | <30/≥30/Missing |  |

^a^ ‘Prior failure on other treatment includes trazodone, nefazodone, bupropion, mirtazapine, mianserin, opipramol, agomelatine, tianeptine, reboxetine, vilazodone and vortioxetine. CGI-S: Clinical Global Impression-Severity; C-SSRS: Columbia-Suicide Severity Rating Scale; EQ-VAS: EuroQoL-visual analogue scale; IPW: inverse probability weighting; MADRS: Montgomery Åsberg Depression Rating Scale; MDD: major depressive disorder; MDE: major depressive episode; MGH-ATRQ: Massachusetts General Hospital Antidepressant Treatment Response Questionnaire; NS: nasal spray; SNRI: serotonin-norepinephrine reuptake inhibitor; SSRI: selective serotonin reuptake inhibitor; TCA: tricyclic antidepressant; TRD: treatment resistant depression.

### Supplementary Table 4. Chance of response at Month 6; esketamine NS vs RW polypharmacy SA

| ITC method | **Predicted probability, %** | | | **OR**  **(95% CI)**  **p value** | **RR**  **(95% CI)**  **p value** | **RD**  **(95% CI)**  **p value** | **NNT**  **(95% CI)** |
| --- | --- | --- | --- | --- | --- | --- | --- |
|  | **(95% CI)** | | |  |  |  |  |
|  | **Esketamine NS** | **RW polypharmacy** | |  |  |  |  |
| Alternative IPW adjustments | | |  |  |  |  |  |
| SA1 (ATC) | 42.7  (38.6–46.8) | | 25.3  (19.6–31.0) | 2.196  (1.555–3.100) <0.0001 | 1.685  (1.320–2.152) <0.0001 | 0.174  (0.103–0.244) <0.0001 | 6  (5–10) |
| SA2 (sATE) | 48.1  (43.8–52.3) | | 26.3  (20.5–32.0) | 2.598  (1.842–3.666) <0.0001 | 1.830  (1.443–2.321) <0.0001 | 0.218  (0.146–0.290) <0.0001 | 5  (4–7) |
| SA3 (ATO) | 46.3  (37.3–55.4) | | 27.1  (19.1–35.2) | 2.317  (1.341–4.004)  0.0026 | 1.707  (1.196–2.436)  0.0032 | 0.192  (0.071–0.313)  0.0019 | 6  (4–15) |
| ATT |  | |  |  |  |  |  |
| Main analysis | 49.7  (45.6–53.9) | 26.8  (21.0–32.5) | | 2.709  (1.930–3.802) <0.0001 | 1.859  (1.474–2.345) <0.0001 | 0.230  (0.159–0.301) <0.0001 | 5  (4–7) |
| SA4 | 43.7  (40.0–47.4) | 25.7  (19.9–31.4) | | 2.247  (1.607–3.142)  <0.0001 | 1.702  (1.341–2.160)  <0.0001 | 0.180  (0.112–0.248)  <0.0001 | 6  (5–9) |
| SA5 | 49.7  (45.6–53.9) | 17.0  (12.2–21.9) | | 4.825  (3.292–7.073)  <0.0001 | 2.923  (2.170–3.937)  <0.0001 | 0.327  (0.263–0.391)  <0.0001 | 4  (3–4) |

All data were calculated using a non-linear mixed model, except ATO. ATO: average treatment effect among the overlap population; CI: confidence interval; IPW: inverse probability weighting; ITC: indirect treatment comparison; NNT: number needed to treat; NS: nasal spray; OR: odds ratio; ATC: average treatment effect among control; ATT: rescaled average treatment effect among treated; RD: risk difference; RR: relative risk; RW: real-world; SA: sensitivity analysis; sATE: stabilised average treatment effect.

### Supplementary Table 5. Chance of remission at Month 6; esketamine NS vs RW polypharmacy SA

| ITC method | **Predicted probability, %** | | | **OR**  **(95% CI)**  **p value** | **RR**  **(95% CI)**  **p value** | **RD**  **(95% CI)**  **p value** | **NNT**  **(95% CI)** |
| --- | --- | --- | --- | --- | --- | --- | --- |
|  | **(95% CI)** | | |  |  |  |  |
|  | **Esketamine NS** | **RW polypharmacy** | |  |  |  |  |
| Alternative IPW adjustments | | |  |  |  |  |  |
| SA1 (ATC) | 28.7  (24.9–32.4) | | 15.6  (10.8–20.3) | 2.184  (1.456–3.276) 0.0002 | 1.845  (1.323–2.571) 0.0003 | 0.131  (0.071–0.192) <0.0001 | 8  (6–15) |
| SA2 (sATE) | 32.5  (28.4–36.5) | | 18.3  (13.2–23.4) | 2.149  (1.461–3.160) 0.0001 | 1.776  (1.310–2.406) 0.0002 | 0.142  (0.077–0.206) <0.0001 | 8  (5–13) |
| SA3 (ATO) | 31.2  (22.8–39.6) | | 16.6  (9.8–23.3) | 2.288  (1.223–4.278)  0.0096 | 0.1886  (1.157–3.073)  0.0109 | 0.147  (0.039–0.254)  0.0077 | 7  (4–26) |
| ATT |  | |  |  |  |  |  |
| Main analysis | 33.6  (29.7–37.6) | 19.4  (14.2–24.6) | | 2.108  (1.449–3.067) 0.0001 | 1.735  (1.297–2.322) 0.0002 | 0.143  (0.078–0.207) <0.0001 | 8  (5–13) |
| SA4 | 29.0  (25.6–32.4) | 18.4  (13.3–23.4) | | 1.817  (1.248–2.646)  0.0019 | 1.580  (1.171–2.132)  0.0028 | 0.107  (0.046–0.168)  0.0006 | 10  (6–22) |
| SA5 | 33.6  (29.7–37.6) | 14.8  (10.2–19.4) | | 2.917  (1.946–4.372)  <0.0001 | 2.272  (1.631–3.166)  <0.0001 | 0.188  (0.128–0.249)  <0.0001 | 6  (5–8) |

All data were calculated using a non-linear mixed model, except ATO. ATO: average treatment effect among the overlap population; CI: confidence interval; IPW: inverse probability weighting; ITC: indirect treatment comparison; NNT: number needed to treat; NS: nasal spray; OR: odds ratio; ATC: average treatment effect among control; ATT: rescaled average treatment effect among treated; RD: risk difference; RR: relative risk; RW: real-world; SA: sensitivity analysis; sATE: stabilised average treatment effect.

### Supplementary Table 6. Baseline characteristics of populations utilised in SA4 and SA5

| **Category**  Mean (SD) unless otherwise stated | **Esketamine NS**  **SA4**  **(N=689)** | **RW polypharmacy**  **SA5**  **(N=230)^a^** |
| --- | --- | --- |
| **Age, years** | 49.4 (12.6) | 51.1 (10.6) |
| **Gender, % (n)**  Female | 63.4 (437) | 61.7 (142) |
| **Age at diagnosis, years** | 34.7 (13.1) | 37.3 (13.2) |
| **Time since first diagnosis of MDD, years** | 14.7 (11.4) | 13.9 (11.4) |
| **Total MADRS score at baseline** | 31.2 (5.0) | 32.2 (6.0) |
| **Total number of failures in current episode** | 2.7 (1.1) | 2.7 (1.0) |
| **CGI-S score** | 4.9 (0.7) | 4.8 (0.8)^b^ |
| **EQ-VAS score** | 44.0 (19.8) | 40.6 (18.1)^c^ |
| **Total number of MDE** | 4.1 (3.6)^d^ | 4.1 (4.3)^d^ |
| **Duration of current MDE, years** | 2.8 (4.6) | 2.4 (2.7) |
| **History of suicidality (based on C-SSRS; lifetime), % (n)**  No event  Suicidal ideation  Suicidal behaviour  Data missing | 59.9 (413)  24.4 (168)  15.7 (108)  0 | 44.8 (103)  30.4 (70)  8.7 (20)  16.1 (37) |
| **Average duration of each treatment line during current MDE, weeks^e^** | 47.4 (73.7) | 46.5 (51.2) |
| **Prior failure on augmentation drug, % (n)** | 17.7 (122) | 15.2 (35) |
| **Prior failure on SSRI, % (n)** | 75.6 (521) | 82.6 (190) |
| **Prior failure on SNRI, % (n)** | 50.9 (351) | 55.2 (127) |
| **Prior failure on TCA, % (n)** | 8.1 (56) | 17.8 (41) |
| **Prior failure on ‘other’ treatments,^f^ % (n)** | 51.5 (355) | 50.4 (116) |

^a^This includes 5 patients who were not included in the main analysis as they had already undergone a treatment switch, combination or augmentation before the Month 6 timepoint. ^b^CGI-S data were missing for one patient on RW polypharmacy; ^c^EQ-VAS data were missing for five patients on RW polypharmacy in SA5; ^d^Data on the number of MDE were missing for one patient on esketamine NS in SA4 and four patients on RW polypharmacy in SA5; ^e^Every patient received multiple treatment lines during their current MDE and these data are the average duration of each individual treatment line; ^f^Prior failure on other treatment includes trazodone, nefazodone, bupropion, mirtazapine, mianserin, opipramol, agomelatine, tianeptine, reboxetine, vilazodone and vortioxetine. CGI-S: Clinical Global Impression-Severity; C-SSRS: Columbia-Suicide Severity Rating Scale; EQ-VAS: EuroQoL-visual analogue scale; MADRS: Montgomery‑Åsberg Depression Rating Scale; MDD: major depressive disorder; MDE: major depressive episode; NS: nasal spray; RW: real-world; SA: sensitivity analysis; SD: standard deviation; SNRI: serotonin-norepinephrine reuptake inhibitor; SSRI: selective serotonin reuptake inhibitor; TCA: tricyclic antidepressant.

### Supplementary Table 7. Threshold analysis based on OR, RR and RD for chance of 6-month response and remission

| **Outcome** | **Efficacy measure** | **Predicted probability,** | | **Difference^b^, %** |
| --- | --- | --- | --- | --- |
|  |  | **% (95% CI)** | |  |
|  |  | **Observed** | **Lowest significant simulated result^a^** |  |
| **Response** |  |  |  |  |
|  | **OR** | 49.7  (45.6–53.9) | 34.2  (30.2–38.1) | 15.6 |
|  | **RD** | 49.7  (45.6–53.9) | 33.8  (29.9–37.7) | 15.9 |
|  | **RR** | 49.7  (45.6–53.9) | 34.3  (30.4–38.3) | 15.4 |
| **Remission** |  |  |  |  |
|  | **OR** | 33.6  (29.7–37.6) | 26.1  (22.5–29.8) | 7.5 |
|  | **RD** | 33.6  (29.7–37.6) | 25.8  (22.1–29.4) | 7.9 |
|  | **RR** | 33.6  (29.7–37.6) | 26.3  (22.6–30.0) | 7.3 |

^a^Pre-determined significance value was p<0.05. ^b^Maximum difference in response/remission before loss of significance in outcomes. CI: confidence interval; OR: odds ratio; RD: risk difference; RR: relative risk.

## Supplementary Figures

### Supplementary Figure 1. Study diagrams for EOTC and SUSTAIN-2

1. SUSTAIN-2 study design

**
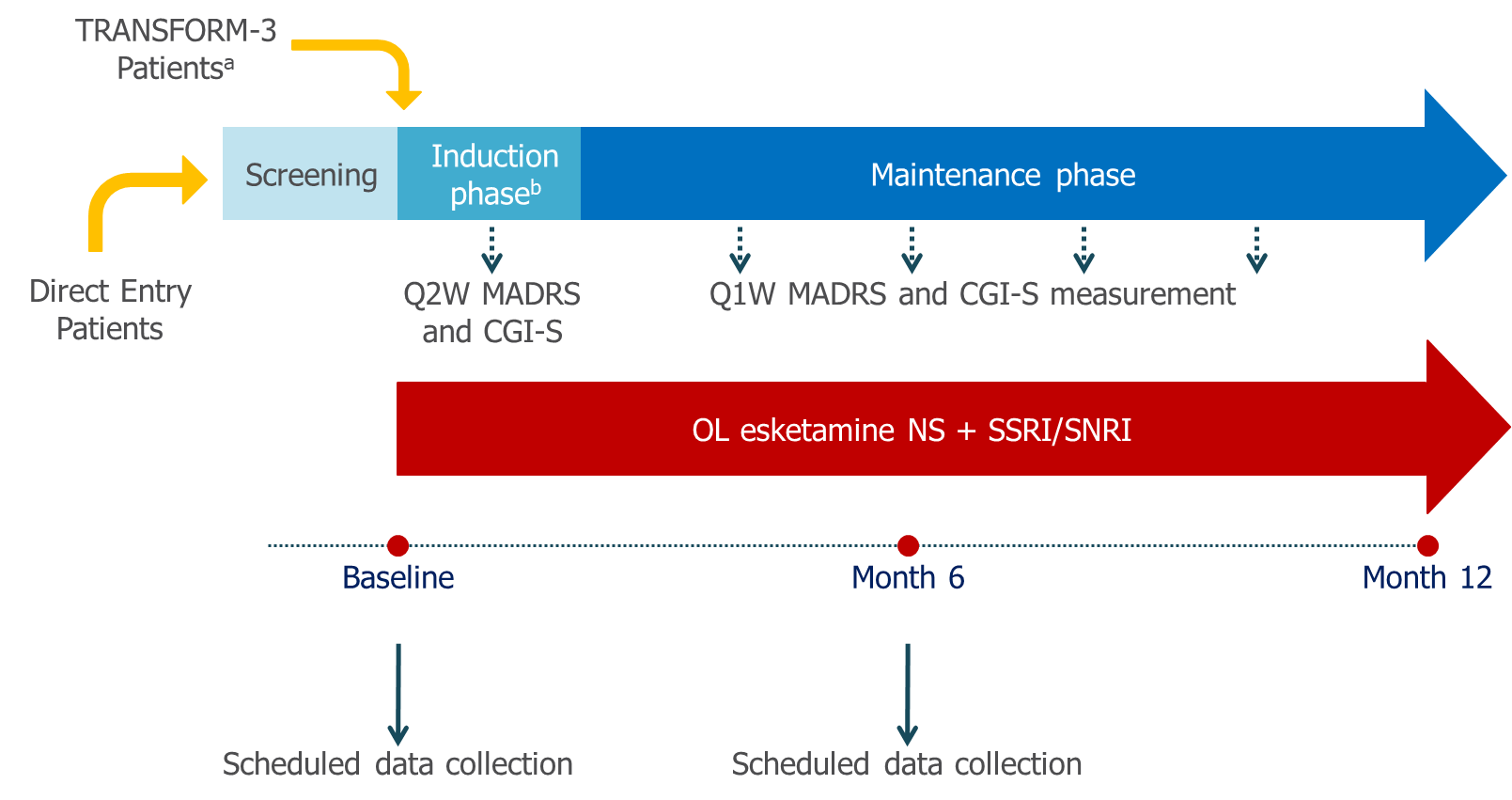
**

1. EOTC study design


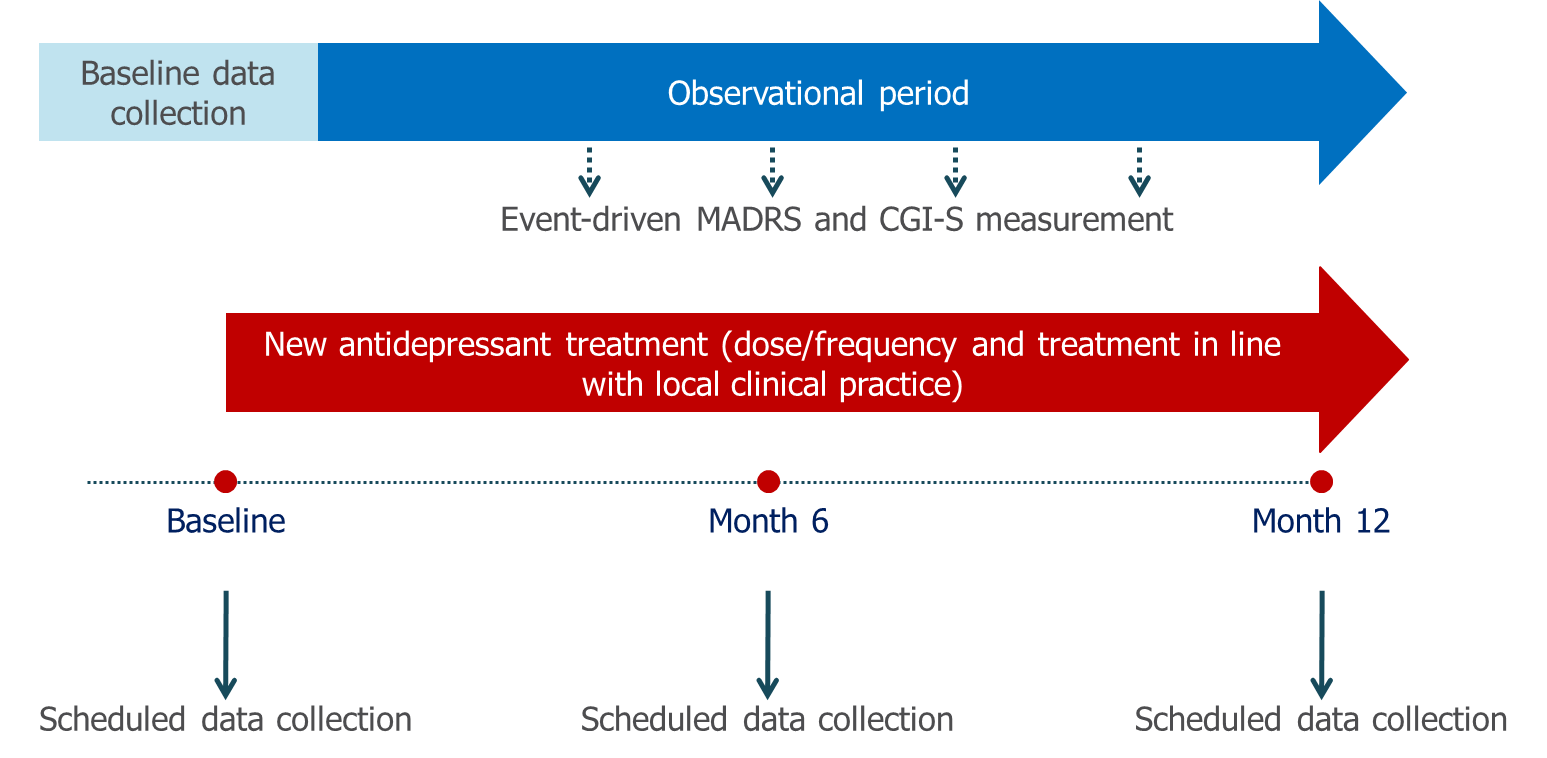


**A**. SUSTAIN-2 study design. **B**. EOTC study design. ^a^Patients from TRANSFORM-3 that were enrolled in the SUSTAIN-2 study were excluded from the ICEBERG analysis as they had already received 4 weeks of treatment with an oral antidepressant plus either esketamine NS or placebo. The EOTC study was terminated when the last enrolled patient reached Month 6; SUSTAIN-2 was terminated when ≥300 and ≥100 patients had received esketamine NS for 6 and 12 months, respectively. When this point was reached, all patients still in SUSTAIN-2 were withdrawn, with “study terminated by sponsor” cited as reason for withdrawal. CGI-S: Clinical Global Impression of Severity; EOTC: European Observational TRD Cohort; MADRS: Montgomery-Åsberg Depression Rating Scale; NS: nasal spray; OL: open label; Q1W: every week; Q2W: every 2 weeks; SNRI: serotonin‑norepinephrine reuptake inhibitor; SSRI: selective serotonin reuptake inhibitor.

### Supplementary Figure 2. Study flow diagrams for patients included in ICEBERG from SUSTAIN-2 and EOTC


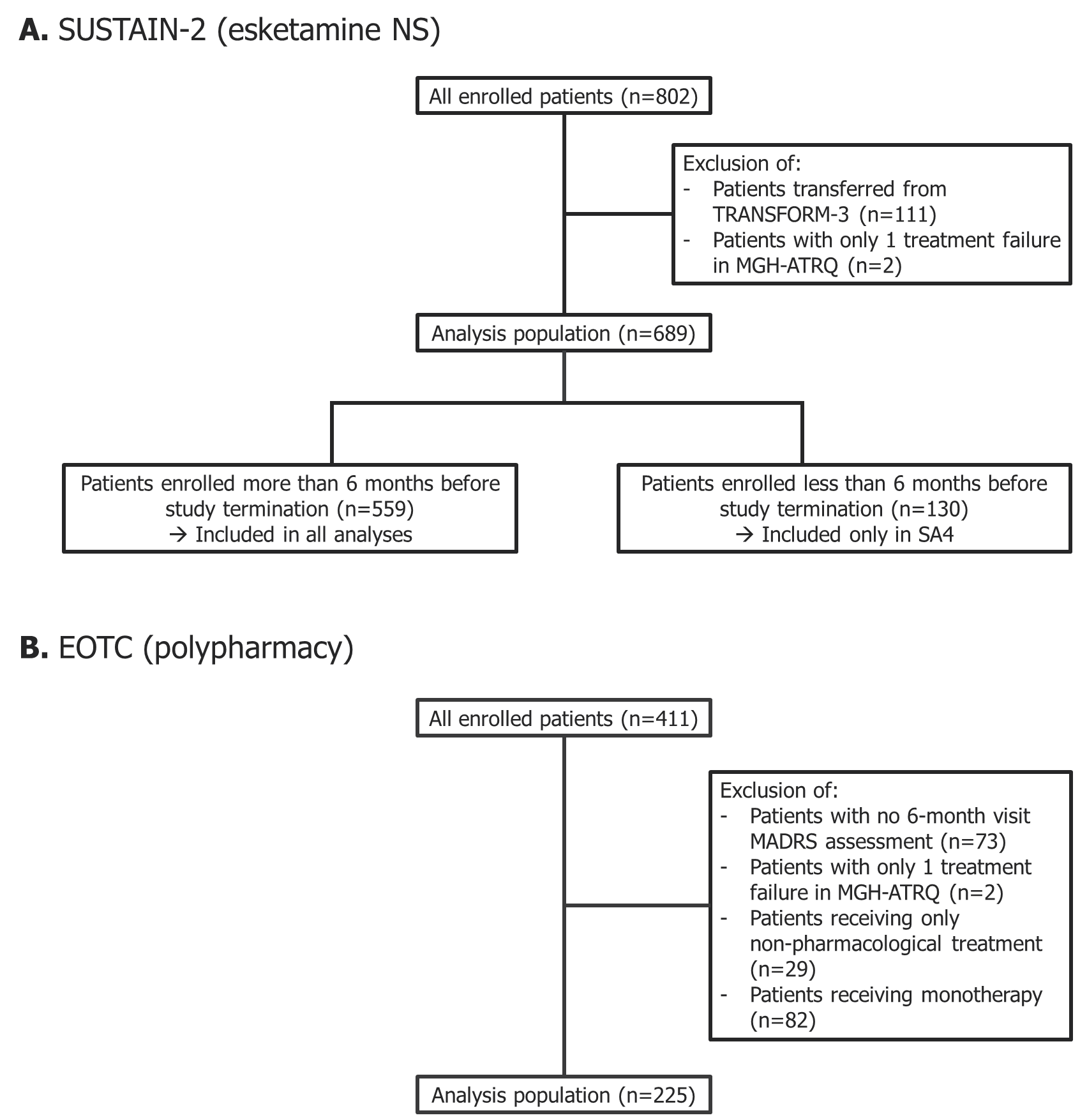


EOTC: European Observational TRD Cohort; MADRS: Montgomery‑Åsberg Depression Rating Scale; MGH-ATRQ: Massachusetts General Hospital Antidepressant Treatment Response Questionnaire; NS: nasal spray; SA: sensitivity analysis.

### Supplementary Figure 3. Effect of PS reweighting on the standardised mean difference between treatments in patients prescribed RW polypharmacy and esketamine NS


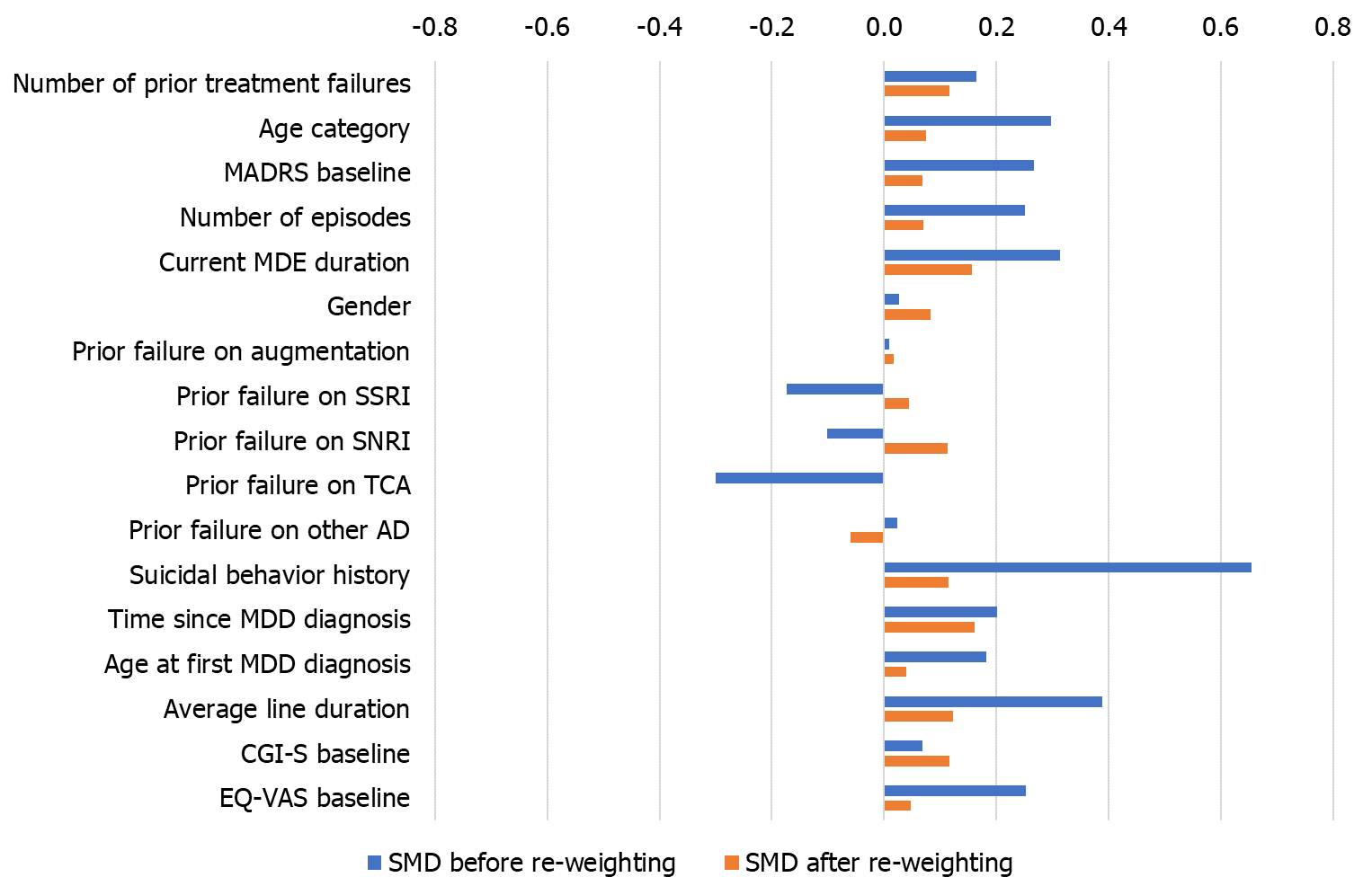


The central vertical line represents esketamine NS data; all other data points represent data from patients prescribed RW polypharmacy, relative to esketamine NS. ‘Prior failure on other AD’ includes trazodone, nefazodone, bupropion, mirtazapine, mianserin, opipramol, agomelatine, tianeptine, reboxetine, vilazodone and vortioxetine. AD: antidepressant; CGI-S: Clinical Global Impression‑Severity; EQ-VAS: EuroQoL‑visual analogue scale; MADRS: Montgomery‑Åsberg Depression Rating Scale; MDD: major depressive disorder; MDE: major depressive episode; NS: nasal spray; RW: real‑world; SMD: standardised mean difference; SNRI: serotonin‑norepinephrine reuptake inhibitor; SSRI: selective serotonin reuptake inhibitor; TCA: tricyclic antidepressant.

### Supplementary Figure 4. Effect of PS reweighting on the standardised mean difference between treatments in patients prescribed RW monotherapy and esketamine NS


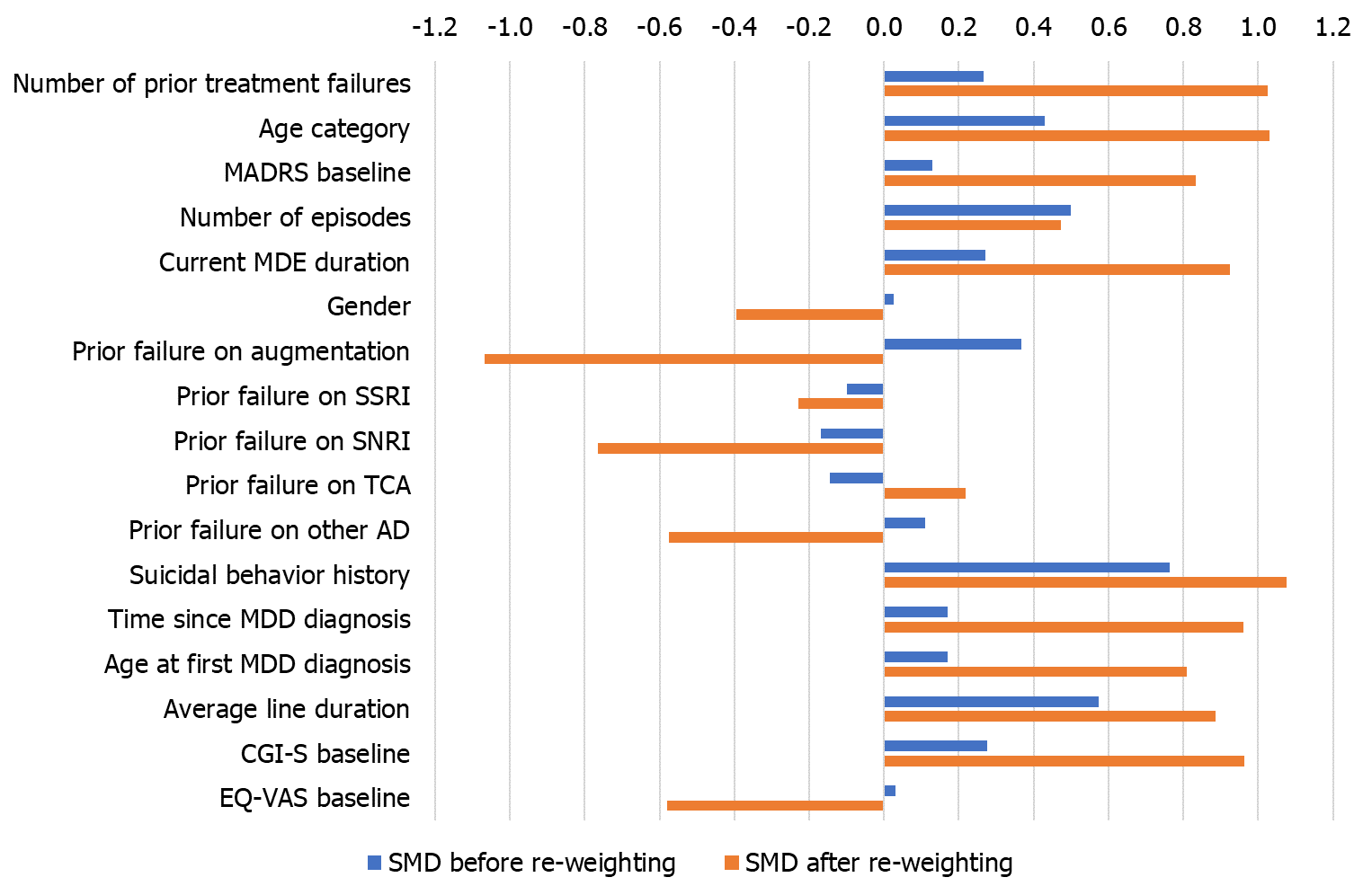


The central vertical line represents esketamine NS data; all other data points represent data from patients prescribed RW polypharmacy, relative to esketamine NS. ‘Prior failure on other AD’ includes trazodone, nefazodone, bupropion, mirtazapine, mianserin, opipramol, agomelatine, tianeptine, reboxetine, vilazodone and vortioxetine. AD: antidepressant; CGI-S: Clinical Global Impression‑Severity; EQ-VAS: EuroQoL‑visual analogue scale; MADRS: Montgomery‑Åsberg Depression Rating Scale; MDD: major depressive disorder; MDE: major depressive episode; NS: nasal spray; RW: real‑world; SMD: standardised mean difference; SNRI: serotonin‑norepinephrine reuptake inhibitor; SSRI: selective serotonin reuptake inhibitor; TCA: tricyclic antidepressant.

### Supplementary Figure 5. Multivariable analysis of response and remission at Month 6

A. Response


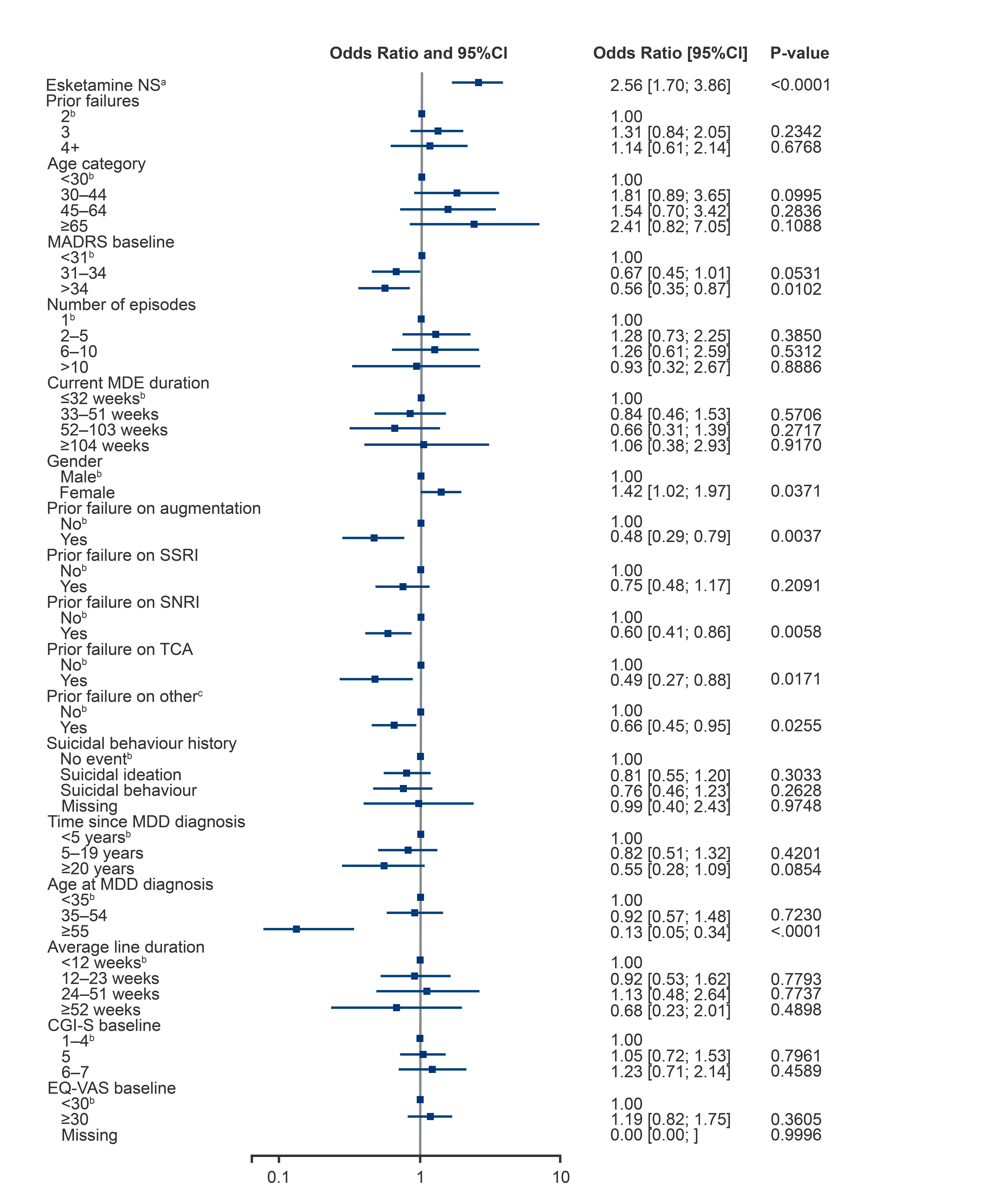


**B.** Remission


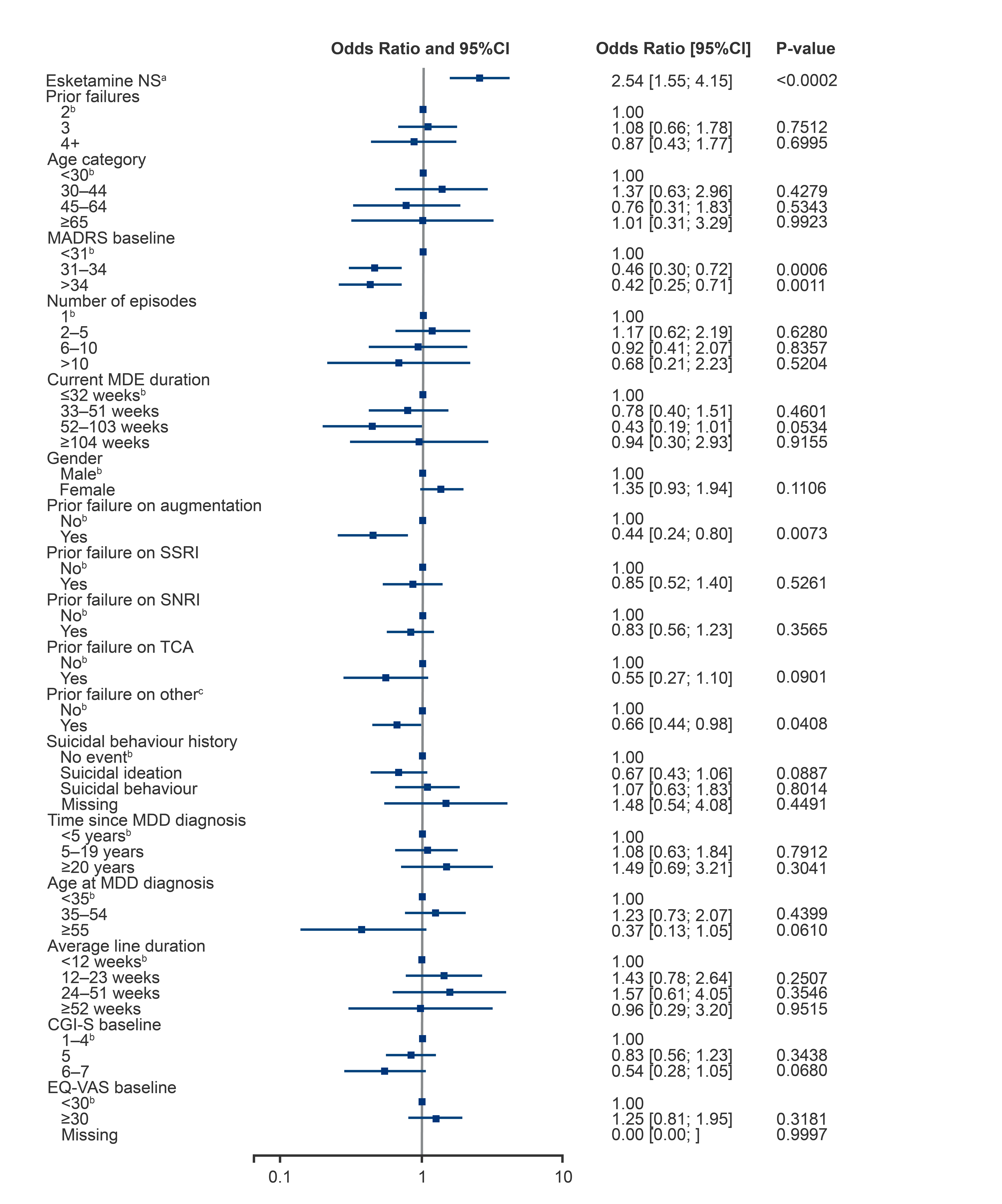


Multivariable logistic regression plots for 6‑month response (A) and remission (B). Individual patient-level data from both studies were pooled. Real‑world treatment excludes esketamine NS. ^a^Given in combination with an SSRI or SNRI; ^b^Reference value; ^c^’Prior failure on other’ includes trazodone, nefazodone, bupropion, mirtazapine, mianserin, opipramol, agomelatine, tianeptine, reboxetine, vilazodone and vortioxetine). CGI-S: Clinical Global Impression – Severity; CI: confidence interval; EQ-VAS: EuroQol Visual Analogue Scale; MADRS: Montgomery-Åsberg Depression Rating Scale; MDD: major depressive disorder; MDE: major depressive episode; NS: nasal spray; OR: odds ratio; SNRI: serotonin-norepinephrine reuptake inhibitor; SSRI: selective serotonin reuptake inhibitor; TCA: tricyclic antidepressant.

# References

American Psychiatric Association, 2013. Diagnostic and Statistical Manual of Mental Disorders, 5^th^ ed. American Psychiatric Association, Arlington, V.A.

Heerlein, K., Young, A.H., Otte, C., Frodl, T., Degraeve, G., Hagedoorn, W., Oliveira-Maia, A.J., Perez Sola, V., Rathod, S., Rosso, G., 2021. Real-world evidence from a European cohort study of patients with treatment resistant depression: baseline patient characteristics. J. Affect. Disord. 283, 115–122.

Müller, M.J., Himmerich, H., Kienzle, B., Szegedi, A., 2003. Differentiating moderate and severe depression using the Montgomery-Asberg depression rating scale (MADRS). J. Affect. Disord. 77(3), 255-260. https//doi:10.1016/s0165-0327(02)00120-9

Wajs, E., Aluisio, L., Holder, R,. Daly, E.J., Lane, R., Lim, P., George, J.E., Morrison, R.L., Sanacora, G., Young, A.H., Kasper, S., Sulaiman, A.H., Li, C.T., Paik, J.W., Manji, H., Hough, D., Grunfeld, J., Jeon, H.J., Wilkinson, S.T., Drevets, W.C., Singh, J.B., 2020. Esketamine Nasal Spray Plus Oral Antidepressant in Patients With Treatment-Resistant Depression: Assessment of Long-Term Safety in a Phase 3, Open-Label Study (SUSTAIN-2). J. Clin. Psych. 81, 19m12891. https//doi:10.4088/JCP.19m12891.

World Health Organisation, 2004. ICD-10: International Statistical Classification of Diseases and Related Health Problems: 10th Revision, 2nd ed. World Health Organization, Geneva.

# Supplementary Video Abstract

<<Ask editors to embed animation here>>
